# Leucine-Enriched Essential Amino Acids Enhance the Antiseizure Effects of the Ketogenic Diet in Rats

**DOI:** 10.3389/fnins.2021.637288

**Published:** 2021-03-19

**Authors:** Fumika Takeuchi, Natsumi Nishikata, Mai Nishimura, Kenji Nagao, Masahito Kawamura

**Affiliations:** ^1^Research Institute for Bioscience Products and Fine Chemicals, Ajinomoto Co., Inc., Kawasaki, Japan; ^2^Department of Pharmacology, Jikei University School of Medicine, Minato-ku, Japan

**Keywords:** epilepsy, adenosine A1 receptors, acute brain slices, hippocampus, *AminoL40*

## Abstract

The classic ketogenic diet (KD) can be used successfully to treat medically refractory epilepsy. However, the KD reduces seizures in 50–70% of patients with medically refractory epilepsy, and its antiseizure effect is limited. In the current study, we developed a new modified KD containing leucine (Leu)-enriched essential amino acids. Compared with a normal KD, the Leu-enriched essential amino acid-supplemented KD did not change the levels of ketosis and glucose but enhanced the inhibition of bicuculline-induced seizure-like bursting in extracellular recordings of acute hippocampal slices from rats. The enhancement of antiseizure effects induced by the addition of Leu-enriched essential amino acids to the KD was almost completely suppressed by a selective antagonist of adenosine A_1_ receptors or a selective dose of pannexin channel blocker. The addition of Leu-enriched essential amino acids to a normal diet did not induce any antiseizure effects. These findings indicate that the enhancement of the antiseizure effects of the KD is mediated by the pannexin channel-adenosine A_1_ receptor pathway. We also analyzed amino acid profiles in the plasma and hippocampus. A normal KD altered the levels of many amino acids in both the plasma and hippocampus. The addition of Leu-enriched essential amino acids to a KD further increased and decreased the levels of several amino acids, such as threonine, histidine, and serine, suggesting that altered metabolism and utilization of amino acids may play a role in its antiseizure effects. A KD supplemented with Leu-enriched essential amino acids may be a new therapeutic option for patients with epilepsy, including medically refractory epilepsy.

## Introduction

The classic ketogenic diet (KD) is a high-fat low-carbohydrate diet therapy designed in the 1920s (Wilder, 1921). The KD induces ketosis and alters metabolism in the brain. This alteration in brain metabolism induces antiseizure effects, and KD can be used successfully to treat patients with pediatric epilepsy. The KD has also been identified as a useful therapy for antiepileptic drug-resistant epilepsy (Hallbook et al., 2007). Despite almost 100 years of clinical use, the mechanisms underlying the antiseizure effects of the KD are not fully understood (Rho and Stafstrom, 2012; Danial et al., 2013; Simeone et al., 2018). In recent decades, several lines of evidence have revealed the molecular mechanisms of the KD, including ATP-sensitive potassium (K_ATP_) channels (Ma et al., 2007; Kawamura et al., 2010), BCL-2-associated agonists of cell death (Gimenez-Cassina et al., 2012), vesicular glutamate transporters (Juge et al., 2010), lactate dehydrogenase (Sada et al., 2015), and adenosine A_1_ receptors (A_1_Rs) (Masino et al., 2011; Kawamura et al., 2014). Although the KD exerts antiseizure effects through various molecular pathways, the efficacy of KD is still limited. It has been reported that the KD causes a 50% reduction in seizures in 50–70% of patients with medically refractory epilepsy (Freeman et al., 2006; Caraballo et al., 2011; Wiemer-Kruel et al., 2017; Kossoff et al., 2018). Therefore, methods to further enhance the efficacy of KD therapy are required for improving the management of epilepsy.

One possible strategy for enhancing the efficacy of the KD therapy is modifying the diet with ketogenic amino acids, such as leucine (Leu). Among the 20 L-amino acids that make up natural proteins, nine are considered nutritionally essential. These essential amino acids may play key roles in regulating brain energy and neurotransmitter metabolism when added to the KD. Branched-chain amino acids (BCAAs), such as Leu, are readily transported across the blood–brain barrier and are also known to play an important role in neurotransmitter metabolism (Hutson et al., 2001). Several studies have assessed the modulatory effects of amino acids on the antiseizure effects of the KD. The addition of BCAAs (Leu:Ile:Val = 9:6:5) to the KD has been reported to reduce seizures in patients with medically refractory epilepsy (Evangeliou et al., 2009). It has been reported that a new combined diet with a low ketogenic ratio (KR) diet composed of specific fatty acids, low-glycemic-index carbohydrates, and high BCAAs protects against seizures to the same extent as the classic KD in a chronic kainate mouse model of epilepsy (Dallerac et al., 2017). In another study, both L- and D-leucine protected mice when administered prior to the onset of seizures induced by kainic aid injection, and D-leucine potently terminated seizures even after the onset of seizure activity (Hartman et al., 2015). These diets use varying combinations of amino acids, and there is very little understanding about the role of these amino acids in the KD. Additionally, when modulating the diet with amino acids, it is important to take overall amino acid balance into consideration because animals detect amino acid balance and alter food intake in response to protein deficiency or excess, and amino acid imbalance causes diet aversion and affects growth (Morrison et al., 2012). Thus, we hypothesized that Leu-enriched essential amino acids, comprising all nine essential amino acids, may enhance the efficacy of the antiseizure effects of KD.

In the current study, we tested two different amino acid mixtures: (1) a Leu-enriched essential amino acid mixture containing 40% Leu, hereinafter referred to as “AminoL40”, and (2) a casein composition amino acid mixture (CaseinAA) as a control. AminoL40 is an amino acid mixture that was formulated to optimize muscle protein synthesis. It has been demonstrated that 1.5 g of AminoL40 stimulates muscle protein synthesis to the same extent as 40 g of whey protein (Wilkinson et al., 2018). Amino acids are categorized into glucogenic amino acids, ketogenic amino acids, or both ketogenic and glucogenic amino acids according to the fate of their degradation products. AminoL40 contains approximately 57% ketogenic amino acids and 27% ketogenic and glucogenic amino acids, while CaseinAA contains only 18% ketogenic amino acids and 20% ketogenic and glucogenic amino acids (Supplementary Figure S1). Ketogenic amino acids are degraded directly into acetyl-CoA, which is the precursor of ketone bodies. The high content of ketogenic amino acids in AminoL40 may be beneficial for sustaining ketosis while providing additional nutritional components for protein synthesis. We used this mixture and compared the effects of AminoL40-supplemented KD and CaseinAA-supplemented KD by biochemical and electrophysiological methods and assessed plasma and hippocampal amino acid profiles.

## Materials and Methods

### Animals

The protocol for these animal experiments was reviewed and approved by the Institutional Animal Care and Use Committee of Jikei University and the Animal Care Committee of Ajinomoto Co., Inc. and conforms to the Guidelines for the Proper Conduct of Animal Experiments of the Science Council of Japan (2006). Sprague–Dawley (SD) rats aged 4–5 weeks were obtained from Sankyo Laboratory Service Co., Ltd. (Shizuoka, Japan). All rats were housed in colony cages and maintained on a 12:12 h light/dark cycle with free access to water.

### Diets

We prepared a CRF-1 diet (Oriental Yeast Co., Ltd., Tokyo, Japan) as a control diet (CD) and four types of KDs: (1) a normal KD without addition of any amino acid (normal KD), (2) a KD with a 3.3% CaseinAA mixture (KD + CA), (3) a KD with a 3.3% AminoL40 mixture (KD + 3.3% AL), and (4) a KD with a 2.5% AminoL40 mixture (KD + 2.5% AL). The compositions of these diets are shown in Supplementary Table S1. The KR was calculated using Woodyatt’s formula (Woodyatt, 1921), which considers the ketogenic macronutrients represented by portions of protein and fat versus glucogenic macronutrients instead of calculating the simple KR using fat: (protein + carbohydrate). Woodyatt’s KR of all types of KD was maintained between 4.8:1 and 5.3:1 (Supplementary Table S1). To evaluate the effect of AminoL40 alone, a non-ketogenic diet based on AIN-93G was prepared with a 3.3% AminoL40 mixture (CD + 3.3% AL). The proportions of each amino acid in the CaseinAA and AminoL40 mixture are shown in Supplementary Table S2.

### Food Intake and Body Weight Measurements and Tissue Sampling

Food intake and body weight were measured twice a week. Sampling of the liver, hippocampus, and blood was performed immediately after treatment under isoflurane anesthesia; the animals were placed in the acrylic induction chamber filled with a mixture of air and isoflurane, and then isoflurane was administered via a mask. The liver and hippocampus were weighed and stored at −80°C until analysis. Blood samples were collected in tubes on ice containing ethylenediaminetetraacetate acid (EDTA). Plasma samples were obtained by centrifuging the blood samples.

### Blood Biochemistry

Blood glucose and ketone body levels in blood samples collected from a small needle stick of the tail vein were measured using FreeStyle Optium Neo blood glucose meters (Abbott Diabetes Care, Witney, Oxfordshire, United Kingdom) and FreeStyle Optium β-ketone Test Strips (Abbott Diabetes Care, Witney, Oxfordshire, United Kingdom). Lactate and insulin concentrations in venous plasma samples were measured using the Lactate Colorimetric Assay Kit II (BioVision Inc., Milpitas, CA, United States) and Morinaga Rat Insulin Measurement Kit (Morinaga Biochemical Research Laboratory, Yokohama, Japan).

### Quantification of Amino Acid and Metabolite Concentrations

The plasma sample was mixed with the internal standard solution (stable isotope-labeled amino acids in water) and deproteinized with acetonitrile. Frozen hippocampal tissue was powdered using a Multi-Beads Shocker (Yasui Kikai, Osaka, Japan) and homogenized in an ice-cold methanol aqueous solution containing L-phenyl-d5-alanine. The homogenate was further mixed with water and chloroform, and the upper phase was dried. The residue was dissolved in water and mixed with the internal standard solution. The plasma and hippocampal samples were derivatized with APDSTAG^®^ (FUJIFILM Wako Pure Chemicals, Osaka, Japan) and analyzed using liquid chromatography coupled with tandem mass spectrometry (LC-MS/MS) as described in a previous publication (Shimbo et al., 2009).

### Extracellular Recordings

Male SD rats aged 4–5 weeks were fed a CD, KD + CA, KD + 2.5% AL, KD + 3.3% AL, or CD + 3.3% AL for 13–16 days. Rats were anesthetized with isoflurane (5% in air) and decapitated. Standard slice preparation was performed, and standard recording conditions were employed for field recordings as described in our previous works (Kawamura et al., 2014; Oyama et al., 2020). Briefly, four to five coronal hippocampal slices of 400-μm thickness were made in ice-cold artificial cerebrospinal fluid (aCSF) containing (in mM) 126 NaCl, 3 KCl, 1.5 MgCl_2_, 2.4 CaCl_2_, 1.2 NaH_2_PO_4_, 11 glucose, and 26 NaHCO_3_ (osmolarity 320 mOsm, pH 7.4 when saturated with 95% O_2_ + 5% CO_2_) using a vibrating slice cutter (PRO 7, Dosaka EM, Kyoto, Japan). The slices were incubated in aCSF saturated with 95% O_2_ + 5% CO_2_ for 30–40 min at 37°C, then kept at room temperature until recording. The slices were then placed on a nylon net in the recording chamber and submerged in and continuously superfused with aCSF at a flow rate of 2 ml min^–1^ at 32 ± 0.5°C using a thermostatic controller (TC-324C, Warner Instruments, Hamden, CT, United States).

Field population spikes (PSs) were recorded using the same method as described in our previous works (Kawamura et al., 2014). Briefly, medium wall (1.5 mm) capillary filament glass was pulled on a Sutter P-97 micropipette puller (Novato, CA, United States), resulting in electrode resistances of 8–12 MΩ. A recording electrode filled with 3 M NaCl was placed in the pyramidal layer of the CA3 region. A twisted, bipolar-insulated tungsten electrode was placed in the hilus of the dentate gyrus as a stimulating electrode; stimuli were delivered at 30 s intervals, the pulse duration was 100 μs, and the intensity was adjusted such that the amplitude of evoked PS responses was between 0.6 and 1.4 mV. Seizure-like activity was induced by blocking GABAergic inhibition with bicuculline (10 μM). KD-induced inhibition of seizure-like activity is sensitive to glucose level (Kawamura et al., 2014). In the current method, the antiseizure effects of acute hippocampal slices were observed by changing extracellular glucose concentration from brain slice-maintenance level (11 mM) to normoglycemic level in the hippocampus (3 mM, Hu and Wilson, 1997). Antiseizure effects were measured by comparing the area under the curve (mV/ms, “area” in Figure 3A) of bicuculline-induced bursting 1 min before and 25 min after changing the concentration of extracellular glucose concentration. PSs were recorded via an AC amplifier (Model 3000, A-M Systems, Carlsborg, WA, United States) and filtered at 1 kHz. The data were digitized (16-channel A/D board, National Instruments Japan, Tokyo, Japan) at a rate of 4 kHz and analyzed online using custom NeuroAcquisition software (Galtware, Denver, CO, United States). All area data of bicuculline-induced bursting were normalized to the baseline (% of baseline) and are expressed as the mean ± standard error (SEM), as described in our previous publication (Kawamura et al., 2014). The graphs in the figures show sparse markers every four points. For the extracellular recordings, the time point indicated as the onset of changing glucose concentration is the calculated time when the solution first began to mix into the aCSF in the slice chamber.

### Drug Applications

Bicuculline, 8-cyclopentyl-1,3-dipropylxanthine (DPCPX) and carbenoxolone (CBX) were purchased from Sigma (St. Louis, MO, United States). As described in our previous publication (Oyama et al., 2020), all drugs were dissolved in aCSF at 100 times the desired final concentration and then applied via a syringe pump (STC-525, Terumo, Tokyo, Japan) to achieve the final concentration. For pretreatment, bicuculline, DPCPX, or CBX was superfused for at least 30 min before changing extracellular glucose. After application of any antagonist, no additional recordings were performed from the same slice.

### Statistical Analysis

The data were compared by using unpaired *t*-tests for two groups and by one-way analysis of variance (ANOVA) with Bonferroni’s correction for three or more groups using GraphPad InStat 3.10 (GraphPad Software, La Jolla, CA, United States) or R 3.6.0 (R Core Team, 2019, R Foundation for Statistical. Computing, Vienna, Austria). *P*-values (*P*) less than 0.05 were considered statistically significant.

## Results

### Body Weight and Blood Biochemistry

**FIGURE 1:**
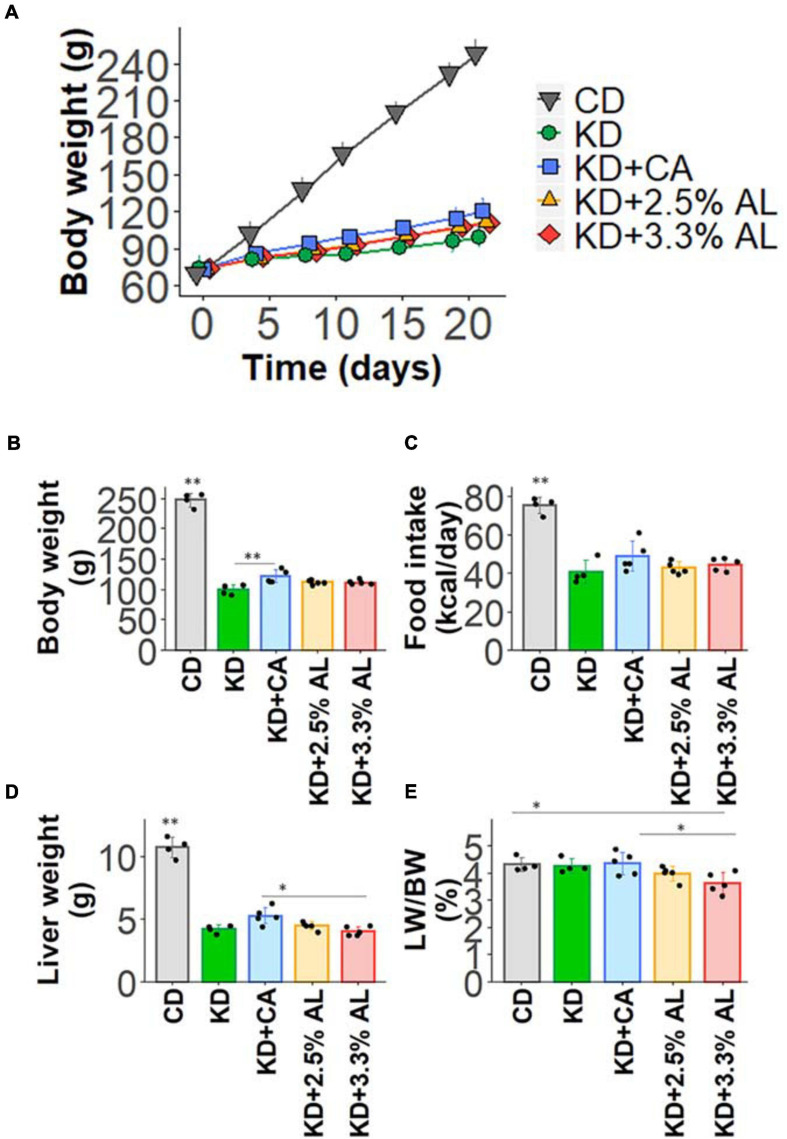
**(A)** Time course of the growth of control diet (CD)-fed, normal ketogenic diet (KD)-fed, KD + CA-fed, KD + 2.5% AL-fed, and KD + 3.3% AL-fed rats. **(B)** Summary of average body weight (g) at 21 days after feeding. *F*(4, 18) = 259.682, *P* < 0.0001; one-way analysis of variance (ANOVA) with *post hoc* test. **(C)** Summary of average food intake (kcal/day) at 21 days after feeding. *F*(4, 18) = 30.934, *P* < 0.0001; one-way ANOVA with *post hoc* test. **(D)** Summary of average liver weight (g) at 21 days after feeding. *F*(4, 18) = 120.589, *P* < 0.0001; one-way ANOVA with *post hoc* test. **(E)** Summary of average liver weight relative to body weight (%). *F*(4, 18) = 3.948, *P* < 0.05; one-way ANOVA with *post hoc* test; CD, *n* = 4; normal KD, *n* = 4; KD + CA, *n* = 5; KD + 2.5% AL, *n* = 5; KD + 3.3% AL, *n* = 5; ^∗^*P* < 0.05; ^∗∗^*P* < 0.01.

**FIGURE 2:**
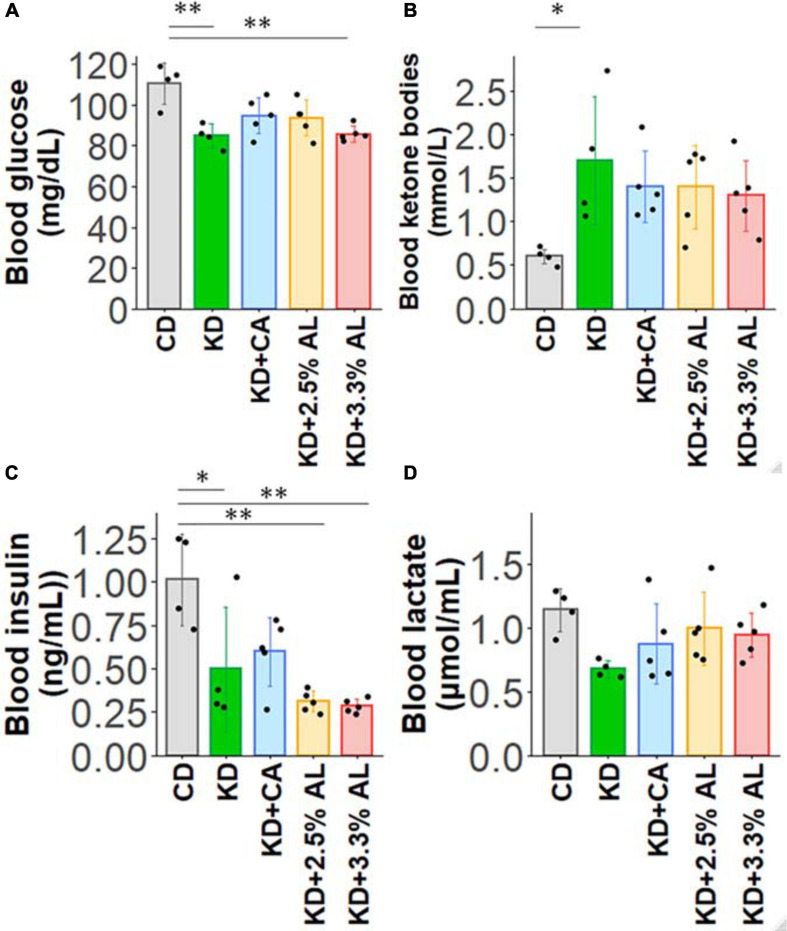
**(A)** Summary of the average blood glucose concentrations (mg/dl) of CD-fed, normal KD-fed, KD + CA-fed, KD + 2.5% AL-fed, and KD + 3.3% AL-fed rats at 21 days after feeding. *F*(4, 18) = 7.274, *P* < 0.01; one-way ANOVA with *post hoc* test. **(B)** Summary of average blood ketone body levels (mmol/L) at 21 days after feeding. *F*(4, 18) = 3.113, *P* < 0.05; one-way ANOVA with *post hoc* test. **(C)** Summary of average blood insulin levels (ng/ml). *F*(4, 18) = 8.7,08, *P* < 0.001; one-way ANOVA with *post hoc* test. **(D)** Summary of average blood lactate levels (μmol/ml). *F*(4, 18) = 2.229, *P* = 0.107; one-way ANOVA with *post hoc* test. CD, *n* = 4; normal KD, *n* = 4; KD + CA, *n* = 5; KD + 2.5% AL, *n* = 5; KD + 3.3% AL, *n* = 5; ^∗^*P* < 0.05; ^∗∗^*P* < 0.01.

We fed a CD, normal KD, KD + CA, KD + 2.5% AL, or KD + 3.3% AL to rats for 2–3 weeks. We did not find that any of the diets had severe adverse effects during this period. We first collected basic data for amino acid-supplemented KD-fed animals. The food intake (kcal/day) of rats fed a normal KD was significantly lower than that of rats fed a CD, but there was no difference among normal the KD, KD + CA, KD + 2.5% AL, and KD + 3.3% AL groups (Figure 1C), indicating that the addition of CaseinAA or AminoL40 to the KD did not affect the food intake of rats. All KD groups exhibited a deceleration of weight gain compared with CD (Figures 1A,B), which is similar to previous findings (Ruskin et al., 2009). The body weight of the KD + CA group was significantly higher than that of the normal KD group at 21 days after diet treatment (Figure 1B) but not different from that of the KD + 2.5% AL or KD + 3.3% AL groups. We also measured the liver weight for each group. The liver weights were significantly lower in the KD groups than in the CD group (Figure 1D), which is also similar to previous findings (Thio et al., 2006). The liver weights corrected for body weight (liver/body weights) of the normal KD, KD + CA, or KD + 2.5% AL group were not different from those of the CD group (Figure 1E), indicating that the reduction in liver weight might have been caused by the deceleration of weight gain. The liver/body weight of the KD + 3.3% AL group was significantly lower than that of the CD and KD + CA groups, but the difference was small (Figure 1E). Compared with CD, blood ketone levels were elevated in normal KD diets (Figure 2B) and was within the range of physiological ketosis observed under a sustained KD (Phillips, 2019). Normal KD group exhibited a significant decrease in blood glucose concentration and a decrease in blood insulin concentration compared with the CD group (Figures 2A,C). The levels of glucose, ketone bodies, and insulin did not differ between the KD, KD + CA, KD + 2.5% AL, and KD + 3.3% AL groups (Figures 2A–C). There was no difference in blood lactate concentrations between any of the groups, including the CD group (Figure 2D). These results suggest that adding amino acids to the KD does not induce significant differences in food intake, body weight, liver weight, or blood biochemistry.

### AminoL40 Enhanced the Antiseizure Effects of Ketogenic Diet

**FIGURE 3:**
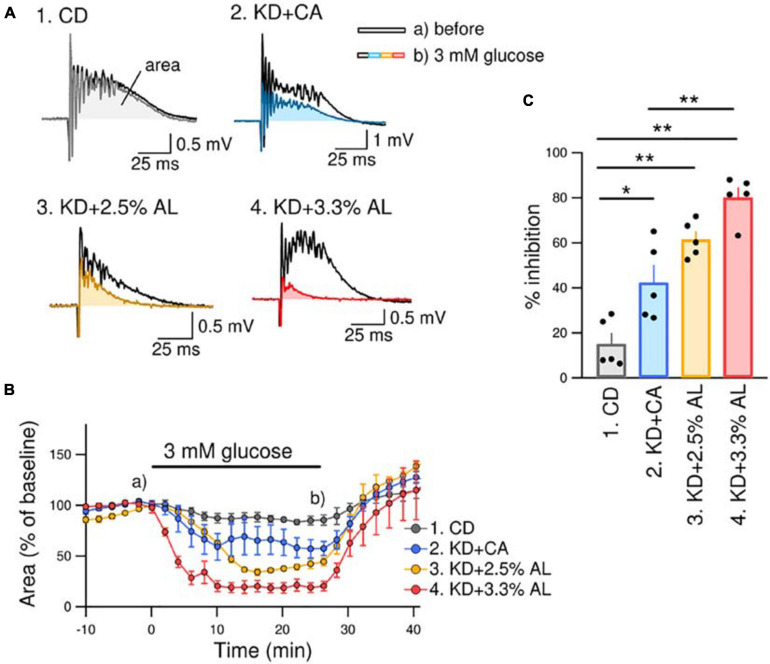
KD + 3.3% AL enhanced the antiseizure effect on bicuculline-induced bursting. **(A)** Traces of bicuculline (10 μM)-induced bursting in acute hippocampal slices from CD-fed (1), KD + CA-fed (2), KD + 2.5% AL-fed (3), and KD + 3.3% AL-fed rats (4). Black traces show seizure-like bursts 1 min before changing the extracellular glucose concentration, and the colored traces show bursting 25 min after changing the extracellular glucose concentration from 11 to 3 mM (3 mM glucose). Both traces were taken at the points indicated in **(B)** (a and b). **(B)** Time course of the effect of 3 mM glucose for 25 min on the area of bicuculline-induced bursting (% of baseline). Averages of the areas are shown for each timepoint every 2 min, and the SEMs are shown as vertical bars. **(C)** Summary of inhibition of the area of seizure-like bursting in 3 mM glucose in slices from CD-fed, KD + CA-fed, KD + 2.5% AL-fed, and KD + 3.3% AL-fed rats. *F*(3, 16) = 27.048, *P* < 0.0001; one-way ANOVA with *post hoc* test; CD, *n* = 5; KD + CA, *n* = 5; KD + 2.5% AL, *n* = 5; KD + 3.3% AL, *n* = 5; *P < 0.05; **P < 0.01.

We investigated the antiseizure effect of AminoL40-supplemented KD. We have previously reported that bicuculline-induced seizure-like bursting in acute hippocampal slices from normal KD-fed rodents is suppressed by reducing the extracellular glucose concentration from brain slice-maintaining levels of glucose (11 mM) to physiological levels of glucose (3 mM) (Kawamura et al., 2014). The effect of the normal KD continued to be observed in acute brain slices for up to 5 h after decapitation. This method is easy and useful for comparing the efficacy of antiseizure effects because it directly measures the inhibition rate of seizure-like bursting. Therefore, we used the same method to compare the antiseizure effects of KD + CA and KD + AL. We recorded PSs in the pyramidal layer of the CA3 region in acute hippocampal slices from rats in all groups. All recorded slices exhibited seizure-like bursting upon bicuculline (10 μM) application. Changing the extracellular glucose concentration from 11 to 3 mM induced inhibition of bicuculline-induced bursting in slices from KD + CA-fed rats but not in those from CD-fed rats (Figure 3). The efficacy of the inhibitory effect of KD + CA on bursting was not different from that of the normal KD (Supplementary Figure S2), indicating that adding CaseinAA to KD does not change the efficacy of the antiseizure effect of KD. KD + 2.5% AL did not significantly change the inhibition rate of bursting suppression compared with KD + CA (Figure 3). However, KD + 3.3% AL significantly enhanced bursting inhibition (Figure 3). These results indicate that 3.3% AL, but not 3.3% CA, enhances the efficacy of the antiseizure effects of KD in acute hippocampal slices from rats.

### Involvement of A_1_R in the Antiseizure Effects of AminoL40-Supplemented Ketogenic Diet

**FIGURE 4:**
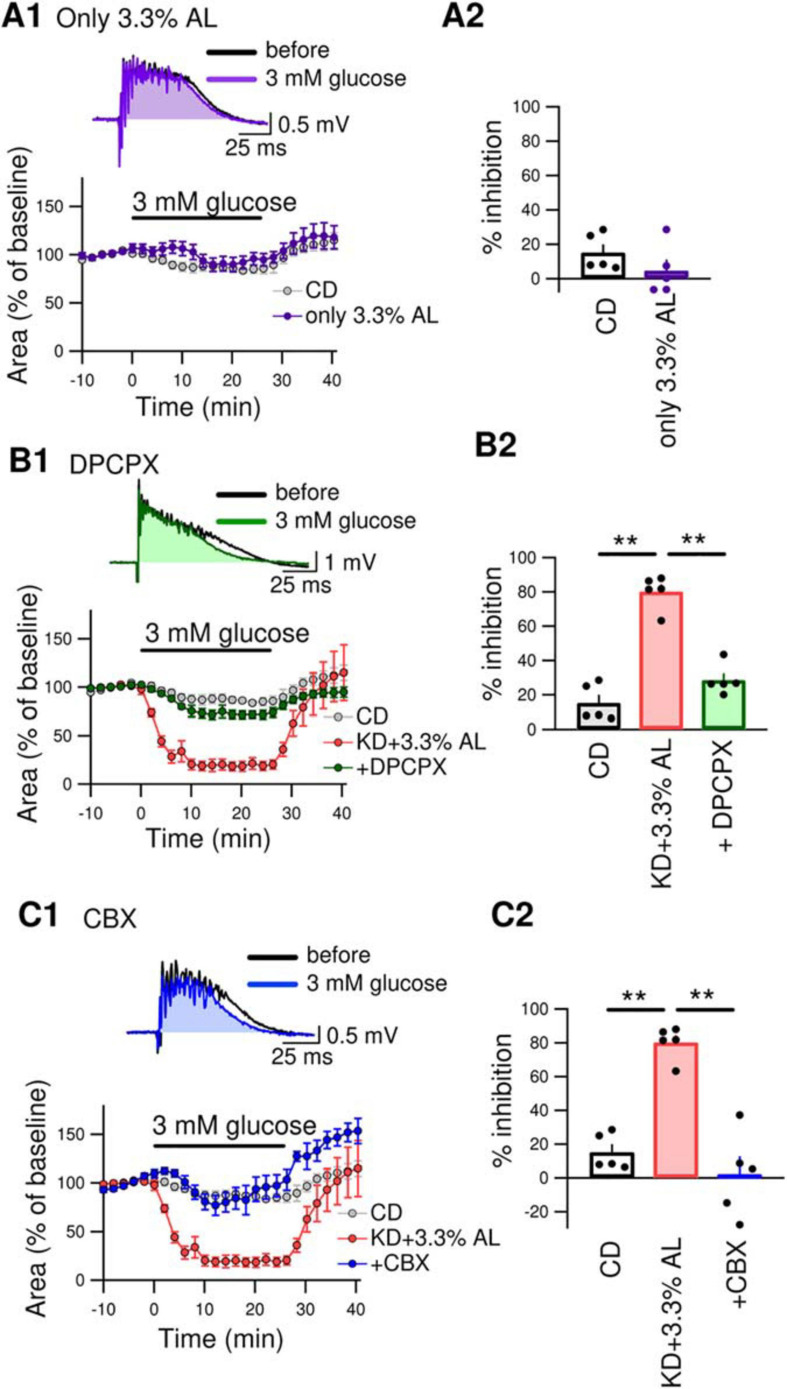
**(A1)** The top traces show bicuculline-induced bursting in acute hippocampal slices from CD + 3.3% AL-fed rats 1 min before and 25 min after changing the extracellular glucose concentration from 11 to 3 mM (3 mM glucose). The time course of the effect of 3 mM glucose for 25min on the area of bicuculline-induced bursting (% of baseline) is shown in the lower graph. **(A2)** Summary of inhibition of the area of seizure-like bursting in slices from CD– and CD + 3.3% AL-fed rats in the presence of 3 mM glucose. *t*(8) = 1.312, *P* = 0.2261; unpaired *t*-test; CD, *n* = 5; CD + 3.3% AL, *n* = 5. The same data for the CD as in Figures 3B,C. **(B1)** The top traces show bursting in acute hippocampal slices from KD + 3.3% AL rats in the presence of 3-dipropylxanthine (DPCPX) (300 nM) 1 min before and 25 min after changing the glucose concentration to 3 mM. The time course of the effect of 3 mM glucose for 25 min on the area of bicuculline-induced bursting (% of baseline) is shown in the lower graph. **(B2)** Summary of inhibition of the area of bursting in the presence of 3 mM glucose. *F*(2, 12) = 61.353, *P* < 0.0001; one-way ANOVA with *post hoc* test; CD, *n* = 5; KD + 3.3% AL, *n* = 5; + DPCPX, *n* = 5; **P < 0.01. The same data for the CD and KD + 3.3% AL as in Figures 3B,C. **(C1)** The top traces show bursting in acute hippocampal slices from KD + 3.3% AL rats in the presence of low-dose carbenoxolone (CBX) (10 μM) 1 min before and 25 min after changing the glucose concentration to 3 mM glucose. The time course of the effect of 3 mM glucose is shown in the lower graph. **(C2)** Summary of inhibition of the area of bursting in the presence of 3 mM glucose. *F*(2, 12) = 31.948, *P* < 0.0001; one-way ANOVA with *post hoc* test; CD, *n* = 5; KD + 3.3% AL, *n* = 5; + CBX, *n* = 5; **P < 0.01. The CD and KD + 3.3% AL data are the same as those in Figures 3B,C.

We next examined whether AminoL40 itself generates any antiseizure effects. A normal diet supplemented with AminoL40 (CD + 3.3% AL) did not induce suppression of bicuculline-induced bursting in hippocampal slices (Figure 4A1–A2), suggesting that AminoL40 itself does not have an antiseizure effect on its own. Previously, we reported that KD-induced inhibition of bursting in hippocampal slices is caused by activation of A_1_Rs following ATP release from pannexin-1 channels and its breakdown to adenosine (Kawamura et al., 2010, 2014). The inhibition of bursting of KD was suppressed by the A_1_R-selective antagonist DPCPX and did not occur in slices from A_1_R-deficient mice. A low dose of CBX (10 μM, which inhibits pannexin channels but not connexin channels) and a pannexin-1-specific peptide antagonist suppressed the inhibition of bursting in slices from KD-fed rats (Kawamura et al., 2014). In the current study, DPCPX (300 nM) significantly suppressed the inhibition of bicuculline-induced bursting in hippocampal slices from KD + 3.3% AL-fed rats (Figure 4B1–B2). A low dose of CBX (10 μM) also suppressed bursting inhibition in hippocampal slices from KD + 3.3% AL-fed rats (Figure 4C1–C2). These results suggest that the enhancement of antiseizure effects by KD + 3.3% AL is mediated by an increase in A_1_R activation through an increase in ATP release from pannexin channels.

### Amino Acid Profiles in the Plasma and Hippocampus

**FIGURE 5:**
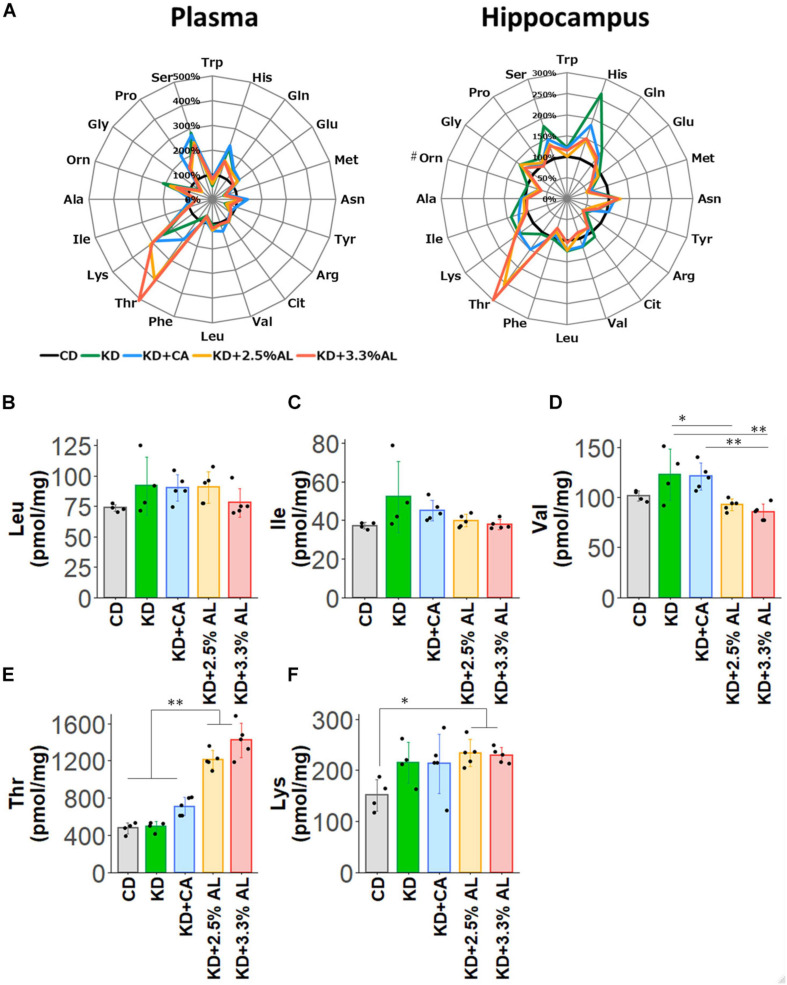
**(A)** Radar charts of amino acid profiles of CD-fed, normal KD-fed, KD + CA-fed, KD + 2.5% AL-fed and KD + 3.3% AL-fed rats at 21 days after feeding. Results are shown for plasma (left) and hippocampus (right). The average levels of amino acids in each diet are compared with the levels in CD-fed and expressed in percentage when CD-fed rats (black) is 100%. #, removing outliers; Trp, tryptophan; His, histidine; Gln, glutamine; Glu, glutamic acid; Met, methionine; Asn, asparagine; Tyr, tyrosine; Arg, arginine; Cit, citrulline; Val, valine; Leu, leucine; BCAAs, branched-chain amino acids; Phe, phenylalanine; Thr, threonine; Lys, lysine; Ile, isoleucine; Ala, alanine; Orn, ornithine; Gly, glycine; Pro, proline; Ser, serine. **(B)** Summary of the average concentration of Leu in the hippocampus (pmol/mg). *F*(4, 18) = 1.614, *P* = 0.214; one-way ANOVA with *post hoc* test. **(C)** Summary of the average concentration of Ile in the hippocampus (pmol/mg). *F*(4, 18) = 2.456, *P* = 0.0829; one-way ANOVA with *post hoc* test. **(D)** Summary of the average concentration of Val in the hippocampus (pmol/mg). *F*(4, 18) = 7.494, *P* < 0.001; one-way ANOVA with *post hoc* test. **(E)** Summary of the average concentration of Thr in the hippocampus (pmol/mg). *F*(4, 18) = 66.991, *P* < 0.0001; one-way ANOVA with *post hoc* test. **(F)** Summary of the average concentration of Lys in the hippocampus (pmol/mg). *F*(4, 18) = 3.362, *P* < 0.05; one-way ANOVA with *post hoc* test. CD, *n* = 4; normal KD, *n* = 4; KD + CA, *n* = 5; KD + 2.5% AL, *n* = 5; KD + 3.3% AL, *n* = 5; ^∗^*P* < 0.05; ^∗∗^*P* < 0.01.

Finally, we examined the amino acid profiles of CD−, normal KD−, KD + CA−, KD + 2.5% AL- and KD + 3.3% AL-fed rats. We measured the concentrations of amino acids and their metabolites in the plasma and hippocampus. Interestingly, compared with the CD, the normal KD altered the levels of many amino acids both in the plasma and hippocampus, generating a distinct amino acid pattern. The addition of CaseinAA to KD did not change the overall amino acid pattern, while further changes were observed upon the addition of AminoL40 (Figure 5A). The levels of all amino acids and metabolites were compared, and compared with the CD, the normal KD increased the mean levels of serine (Ser), asparagine (Asn), histidine (His), lysine (Lys), proline (Pro), ornithine (Orn), glutamine (Gln), and aABA in the plasma (Supplementary Table S3) and the levels of Ser, Asn, His, Lys, Gln, glycine (Gly), Car, cystathionine (Cysthi), and alpha-aminobutyric acid (aABA) in the hippocampus (Supplementary Table S4) and decreased the levels of tryptophan (Trp), methionine (Met), tyrosine (Tyr), phenylalanine (Phe), Gly, alanine (Ala), cystine (Cys2), hydroxyproline, and taurine (Tau) in the plasma (Supplementary Table S3) and the levels of Met, arginine (Arg), and hydroxyproline in the hippocampus (Supplementary Table S4). Compared with the normal KD, both KD + 2.5% AL and KD + 3.3% AL increased the mean levels of threonine (Thr) in the plasma and the levels of Thr and carnitine (Car) in the hippocampus (Figure 5E) and decreased the mean levels of His, Ser, and sarcosine (Sar) in the plasma and the levels of His, Ser, Gln, valine (Val), and aABA in the hippocampus (Supplementary Table S4). At the higher dose of AminoL40 (3.3%), additional changes were observed: increased levels of Trp, decreased levels of Ser and His in the plasma, and decreased mean levels of Phe in the hippocampus (Supplementary Table S4). Despite the additional intake of BCAAs included in AminoL40, rats fed KD + 2.5% AL and KD + 3.3% AL did not exhibit increased plasma and hippocampal levels of Leu, isoleucine (Ile), Val, and Lys compared with those fed the KD (Figures 5B–F).

## Discussion

In the current study, we developed a new modified KD containing leucine (Leu)-enriched essential amino acids (AminoL40). We compared the inhibitory effect of each ketogenic diet on bicuculline-induced seizure-like bursting as well as blood chemistry and amino acid profiles. Compared with normal KD, AminoL40-supplemented KD (KD + 3.3% AL) did not change blood biochemistry. However, AminoL40-supplemented KD increased the inhibition rate of seizure-like bursting in acute hippocampal slices. The inhibitory effect of the AminoL40-supplemented KD on bursting was almost completely suppressed by DPCPX and by a low dose of CBX. The addition of AminoL40 to a normal diet did not inhibit bursting, suggesting that AminoL40 itself has no antiseizure effect and that AminoL40 enhances antiseizure effects of the KD through the pannexin-A_1_R antiseizure pathway.

The ketogenic diet is widely acknowledged as effective in treating epilepsy, and many patients with drug-resistant seizures benefit from this non-pharmacologic option. However, its efficacy is still limited. It has been reported that the KD causes a 50% reduction in seizures in 50–70% of patients with medically refractory epilepsy (Freeman et al., 2006; Caraballo et al., 2011; Wiemer-Kruel et al., 2017; Kossoff et al., 2018). In the current study, we also found that normal KD inhibited seizure-like bursting only by half (Figure 3), indicating that KD alone achieves only limited antiseizure effects. We found that adding AminoL40 to the KD enhanced the antiseizure effects of the KD.

Activation of adenosine receptors is one of the key mechanisms of KD-induced antiseizure effects. The KD has been reported to reduce the frequency and duration of electrographic seizures in mice with genetically induced seizures. Such reduction is suppressed by intraperitoneal injection of the A_1_R selective antagonist DPCPX, and KD has no effect on A_1_R-deficient mice (Masino et al., 2011). It has also been reported that exogenous ketone supplementation with ketone esters or ketone salts induces anticonvulsant effects through activation of A_1_R (Kovacs et al., 2017). These *in vivo* studies show that KD promotes A_1_R-induced antiseizure effects. We also reported the antiseizure effects of A_1_R in an *in vitro* study (Kawamura et al., 2014). KD is known to increase brain ATP levels in both humans (Pan et al., 1999) and rodents (DeVivo et al., 1978; Nakazawa et al., 1983; Nylen et al., 2009), and the source of adenosine may be such increase in ATP levels. Our previous report showed that when the intracellular ATP concentration is increased in hippocampal CA3 pyramidal neurons, physiological levels of extracellular glucose open pannexin-1 channels and release intracellular ATP into the extracellular space. Released ATP is rapidly broken down to adenosine, and adenosine activates A_1_R leading to subsequent opening of K_ATP_ channels. Opening of K_ATP_ channels promotes hyperpolarization and reduces the excitability of CA3 pyramidal neurons (Kawamura et al., 2010). These reports indicate that one of the mechanisms of KD-induced antiseizure effects is the pannexin-1 channel–A_1_R-K_ATP_ channel pathway and involves an increase in brain ATP levels. In the current study, we found that KD + 3.3% AL-induced inhibition of bursting was almost completely suppressed by a selective A_1_R antagonist or the selective dose of pannexin inhibitor CBX. In the absence of the KD, AminoL40 did not induce any antiseizure effects. These findings suggest that improved efficacy of KD by AminoL40 is induced by increased activation of A_1_R through ATP release from pannexin-1 channels.

Adenosine receptors are well established to have therapeutic potential for neurological disorders, including epilepsy, stroke, neuropathic pain, and mental disorders (Masino et al., 2009). Recent evidence shows that the KD improves the behaviors of animal models of neuropathic pain (Ruskin et al., 2009), autism spectrum disorder (Ruskin et al., 2013), and schizophrenia (Kraeuter et al., 2015). Enrichment with AminoL40 might enhance the therapeutic effects of the KD for neurological disorders such as autism spectrum disorder, which is often comorbid with epilepsy.

We found that the addition of AminoL40 enhanced the antiseizure effects of the KD through activation of A_1_R. However, the detailed mechanism regarding how the essential amino acids in AminoL40 trigger the pannexin-1 channel-A_1_R pathway when added to the KD is still undetermined. Our new amino acid mixture, AminoL40, contains 40% Leu along with other essential amino acids, which are known to play key roles in brain energy and neurotransmitter metabolism. Amino acids can be metabolized to provide tricarboxylic acid (TCA) cycle intermediates and to replenish the cycle through anaplerosis (Owen et al., 2002; Sonnewald, 2014; Sperringer et al., 2017). Leu is oxidized to acetyl-CoA through acetoacetate and utilized in the TCA cycle, which is the only known pathway for the terminal oxidation of Leu. Other essential amino acids are similarly metabolized into compounds such as acetyl-CoA, succinyl-CoA, and fumarate, which can enter the TCA cycle and provide carbon skeletons (Supplementary Figure S3; Berg et al., 2002). During starvation, Gln is converted to glutamic acid (Glu) and then to α-ketoglutarate and enters the TCA cycle, which leads to energy production (Owen et al., 2002). Additionally, Leu can activate glutamate dehydrogenase and enhance the conversion of Glu to α-ketoglutarate, which is the rate-determining step of the TCA cycle (Wang et al., 2018; Biswas et al., 2019). Therefore, under KD feeding, which mimics starvation, amino acids may have a large effect on fueling the TCA cycle.

We assessed amino acid profiles and found that compared with a normal diet, the normal KD significantly altered both plasma and hippocampal amino acid levels. All KD groups showed elevated mean levels of Ser, Asn, His, Lys, Pro, and Gln in the plasma and elevated mean levels of Ser, Asn, His, Lys, Gln, and Gly in the hippocampus. Each of these amino acids can provide carbon skeletons for different TCA cycle intermediates. The addition of AminoL40 to the KD induced further changes in the plasma and hippocampal levels of some amino acids. Surprisingly, there were no significant increases in BCAA levels in the plasma and hippocampus, even following the addition of AminoL40. It is possible that amino acids such as BCAAs are used as energy sources in the brain as well as peripheral muscles under low-glucose conditions and that changes in amino acid profiles could not be detected. We found that Thr levels strongly correlated with the Thr content ratio in each diet, and the KD + 3.3% AL group showed significantly higher Thr levels than that of other groups. Threonine dehydratase catabolizes Thr to 2-ketobutyrate, which is converted to succinyl-CoA. When levels are increased, Thr might enter the TCA cycle through the threonine dehydratase pathway and might alter ATP synthesis. The addition of a specific combination of amino acids may have supported the production of energy by replenishing TCA cycle intermediates via anaplerotic reactions, thus leading to an increase in ATP synthesis and enhancing ATP release through pannexin channels, which may have induced antiseizure effects through A_1_R. Further studies, such as flux analysis studies, are needed to clarify the detailed mechanism of action of AminoL40 in improving brain energy metabolism.

In the current study, we found that AminoL40 enhanced the antiseizure effects of the KD. AminoL40 is composed of nine essential amino acids. Administration of AminoL40-supplemented diets for the experimental period resulted in no severe adverse effects and did not change the basic peripheral effects of the normal KD, suggesting that AminoL40 can be used safely in rodents. Additional studies are needed in the future to clarify the effect of Leu-enriched essential amino acids in ketogenic diets used in clinical settings. Leu-enriched essential amino acids may provide new options for the KD treatment of epilepsy, including medically refractory epilepsy.

## Data Availability Statement

The original contributions presented in the study are included in the article/Supplementary Material, further inquiries can be directed to the corresponding author/s.

## Ethics Statement

The animal study was reviewed and approved by the Institutional Animal Care and Use Committee of Jikei University and the Animal Care Committee of Ajinomoto Co., Inc.

## Author Contributions

NN, KN, and MK conceived, designed the research, edited, and revised the manuscript. FT, MN, and MK performed the experiments and analyzed the data. FT and MK prepared the figures. FT, NN, KN, and MK drafted the manuscript. FT, NN, MN, KN, and MK approved the final version of manuscript. All authors contributed to the article and approved the submitted version.

## Conflict of Interest

FT, NN, MN, and KN were employees of Ajinomoto Co., Inc. The remaining author declares that the research was conducted in the absence of any commercial or financial relationships that could be construed as a potential conflict of interest.

## Abbreviations

KD: ketogenic diet
CD: control diet
BCAAs: branched-chain amino acids
TCA cycle: tricarboxylic acid cycle
A_1_R: adenosine A_1_ receptor
K_ATP_ channels: ATP-sensitive potassium channels
DPCPX: 8-cyclopentyl-1,3-dipropylxanthine
CBX: carbenoxolone
Trp: tryptophan
His: histidine
Gln: glutamine
Glu: glutamic acid
Met: methionine
Asn: asparagine
Tyr: tyrosine
Arg: arginine
Cit: citrulline
Val: valine
Leu: leucine
Phe: phenylalanine
Thr: threonine
Lys: lysine
Ile: isoleucine
Ala: alanine
Orn: ornithine
Gly: glycine
Pro: proline
Ser: serine
α AAA: α-aminoadipic acid
α ABA: α-aminobutyric acid
Ala: alanine
Asp: aspartic acid
Car: carnitine
Cysthi: cystathionine
GABA: gamma-aminobutyric acid
HyPro: hydroxyproline
Sar: sarcosine
Tau: taurine
Cys2: cystine
1-MeHis: 1-methylhistidine
3-MeHis: 3-methylhistidine.

## References

[B1] Berg J. M. Tymoczko J. L. Stryer L. (2002). *Biochemistry.* New York, Ny: W.H. Freeman.

[B2] Biswas D. Duffley L. Pulinilkunnil T. (2019). Role of branched-chain amino acid-catabolizing enzymes in intertissue signaling, metabolic remodeling, and energy homeostasis. *FASEB J.* 33 8711–8731. 10.1096/fj.201802842RR 31084571

[B3] Caraballo R. Vaccarezza M. Cersosimo R. Rios V. Soraru A. Arroyo H. (2011). Long-term follow-up of the ketogenic diet for refractory epilepsy: multicenter argentinean experience in 216 pediatric patients. *Seizure* 20 640–645. 10.1016/j.seizure.2011.06.009 21763159

[B4] Dallerac G. Moulard J. Benoist J. F. Rouach S. Auvin S. Guilbot A. (2017). Non-ketogenic combination of nutritional strategies provides robust protection against seizures. *Sci. Rep.* 7:5496. 10.1038/s41598-017-05542-3 28710408 PMC5511156

[B5] Danial N. N. Hartman A. L. Stafstrom C. E. Thio L. L. (2013). How does the ketogenic diet work? Four potential mechanisms. *J. Child Neurol.* 28 1027–1033. 10.1177/0883073813487598 23670253 PMC3971996

[B6] DeVivo D. C. Leckie M. P. Ferrendelli J. S. McDougal D. B. Jr. (1978). Chronic ketosis and cerebral metabolism. *Ann. Neurol.* 3 331–337. 10.1002/ana.410030410 666275

[B7] Evangeliou A. Spilioti M. Doulioglou V. Kalaidopoulou P. Ilias A. Skarpalezou A. (2009). Branched chain amino acids as adjunctive therapy to ketogenic diet in epilepsy: pilot study and hypothesis. *J. Child Neurol.* 24 1268–1272. 10.1177/0883073809336295 19687389

[B8] Freeman J. Veggiotti P. Lanzi G. Tagliabue A. Perucca E. (2006). The ketogenic diet: from molecular mechanisms to clinical effects. *Epilepsy Res.* 68 145–180. 10.1016/j.eplepsyres.2005.10.003 16523530

[B9] Gimenez-Cassina A. Martinez-Francois J. R. Fisher J. K. Szlyk B. Polak K. Wiwczar J. (2012). BAD-dependent regulation of fuel metabolism and K(ATP) channel activity confers resistance to epileptic seizures. *Neuron* 74 719–730. 10.1016/j.neuron.2012.03.032 22632729 PMC3361694

[B10] Hallbook T. Kohler S. Rosen I. Lundgren J. (2007). Effects of ketogenic diet on epileptiform activity in children with therapy resistant epilepsy. *Epilepsy Res.* 77 134–140. 10.1016/j.eplepsyres.2007.09.008 17996423

[B11] Hartman A. L. Santos P. O’Riordan K. J. Stafstrom C. E. Hardwick J. M. (2015). Potent anti-seizure effects of D-leucine. *Neurobiol. Dis.* 82 46–53. 10.1016/j.nbd.2015.05.013 26054437 PMC4640989

[B12] Hu Y. Wilson G. S. (1997). Rapid changes in local extracellular rat brain glucose observed with an in vivo glucose sensor. *J. Neurochem.* 68 1745– 1752. 9084449 10.1046/j.1471-4159.1997.68041745.x

[B13] Hutson S. M. Lieth E. LaNoue K. F. (2001). Function of leucine in excitatory neurotransmitter metabolism in the central nervous system. *J. Nutr.* 131 846S–850S. 10.1093/jn/131.3.846S 11238772

[B14] Juge N. Gray J. A. Omote H. Miyaji T. Inoue T. Hara C. (2010). Metabolic control of vesicular glutamate transport and release. *Neuron* 68 99–112. 10.1016/j.neuron.2010.09.002 20920794 PMC2978156

[B15] Kawamura M. Jr. Ruskin D. N. Geiger J. D. Boison D. Masino S. A. (2014). Ketogenic diet sensitizes glucose control of hippocampal excitability. *J. Lipid Res.* 55 2254–2260. 10.1194/jlr.M046755 25170119 PMC4617128

[B16] Kawamura M. Jr. Ruskin D. N. Masino S. A. (2010). Metabolic autocrine regulation of neurons involves cooperation among pannexin hemichannels, adenosine receptors, and KATP channels. *J. Neurosci.* 30 3886–3895. 10.1523/JNEUROSCI.0055-10.2010 20237259 PMC2872120

[B17] Kossoff E. H. Zupec-Kania B. A. Auvin S. Ballaban-Gil K. R. Christina Bergqvist A. G. Blackford R. (2018). Optimal clinical management of children receiving dietary therapies for epilepsy: updated recommendations of the International Ketogenic Diet Study Group. *Epilepsia Open* 3 175–192. 10.1002/epi4.12225 29881797 PMC5983110

[B18] Kovacs Z. D’Agostino D. P. Dobolyi A. Ari C. (2017). Adenosine A1 receptor antagonism abolished the anti-seizure effects of exogenous ketone supplementation in wistar albino Glaxo Rijswijk rats. *Front. Mol. Neurosci.* 10:235. 10.3389/fnmol.2017.00235 28790891 PMC5524776

[B19] Kraeuter A. K. Loxton H. Lima B. C. Rudd D. Sarnyai Z. (2015). Ketogenic diet reverses behavioral abnormalities in an acute NMDA receptor hypofunction model of schizophrenia. *Schizophr. Res.* 169 491–493. 10.1016/j.schres.2015.10.041 26547882

[B20] Ma W. Berg J. Yellen G. (2007). Ketogenic diet metabolites reduce firing in central neurons by opening K(ATP) channels. *J. Neurosci.* 27 3618–3625. 10.1523/JNEUROSCI.0132-07.2007 17409226 PMC6672398

[B21] Masino S. A. Kawamura M. Wasser C. A. Pomeroy L. T. Ruskin D. N. (2009). Adenosine, ketogenic diet and epilepsy: the emerging therapeutic relationship between metabolism and brain activity. *Curr. Neuropharmacol.* 7 257–268. 10.2174/157015909789152164 20190967 PMC2769009

[B22] Masino S. A. Li T. Theofilas P. Sandau U. S. Ruskin D. N. Fredholm B. B. (2011). A ketogenic diet suppresses seizures in mice through adenosine A(1) receptors. *J. Clin. Invest.* 121 2679–2683. 10.1172/JCI57813 21701065 PMC3223846

[B23] Morrison C. D. Reed S. D. Henagan T. M. (2012). Homeostatic regulation of protein intake: in search of a mechanism. *Am. J. Physiol. Regul. Integr. Comp. Physiol.* 302 R917–R928. 10.1152/ajpregu.00609.2011 22319049 PMC3330767

[B24] Nakazawa M. Kodama S. Matsuo T. (1983). Effects of ketogenic diet on electroconvulsive threshold and brain contents of adenosine nucleotides. *Brain Dev.* 5 375–380. 6638394 10.1016/s0387-7604(83)80042-4

[B25] Nylen K. Velazquez J. L. Sayed V. Gibson K. M. Burnham W. M. (2009). The effects of a ketogenic diet on ATP concentrations and the number of hippocampal mitochondria in Aldh5a1(-/-) mice. *Biochim. Biophys. Acta* 1790 208–212. 10.1016/j.bbagen.2008.12.005 19168117 PMC2646796

[B26] Owen O. E. Kalhan S. C. Hanson R. W. (2002). The key role of anaplerosis and cataplerosis for citric acid cycle function. *J. Biol. Chem.* 277 30409–30412. 10.1074/jbc.R200006200 12087111

[B27] Oyama Y. Ono K. Kawamura M. Jr. (2020). Mild hypothermia protects synaptic transmission from experimental ischemia through reduction in the function of nucleoside transporters in the mouse hippocampus. *Neuropharmacology* 163:107853. 10.1016/j.neuropharm.2019.107853 31734385

[B28] Pan J. W. Bebin E. M. Chu W. J. Hetherington H. P. (1999). Ketosis and epilepsy: 31P spectroscopic imaging at 4.1 T. *Epilepsia* 40 703–707. 10368066 10.1111/j.1528-1157.1999.tb00766.x

[B29] Phillips M. C. L. (2019). “Ketogenic diet therapies in children and adults with epilepsy,” in *Epilepsy - Advances in Diagnosis and Therapy*, ed. Al-Zwaini I. J. (London: IntechOpen).

[B30] R Core Team (2019). *R: A Language and Environment for Statistical Computing.* Vienna: R Foundation for Statistical Computing. Available online at: https://www.R-project.org/

[B31] Rho J. M. Stafstrom C. E. (2012). The ketogenic diet: what has science taught us? *Epilepsy Res.* 100 210–217. 10.1016/j.eplepsyres.2011.05.021 21856126

[B32] Ruskin D. N. Kawamura M. Masino S. A. (2009). Reduced pain and inflammation in juvenile and adult rats fed a ketogenic diet. *PLoS One* 4:e8349. 10.1371/journal.pone.0008349 20041135 PMC2796387

[B33] Ruskin D. N. Svedova J. Cote J. L. Sandau U. Rho J. M. Kawamura M. (2013). Ketogenic diet improves core symptoms of autism in BTBR mice. *PLoS One* 8:e65021. 10.1371/journal.pone.0065021 23755170 PMC3673987

[B34] Sada N. Lee S. Katsu T. Otsuki T. Inoue T. (2015). Epilepsy treatment. Targeting LDH enzymes with a stiripentol analog to treat epilepsy. *Science* 347 1362–1367. 10.1126/science.aaa1299 25792327

[B35] Shimbo K. Oonuki T. Yahashi A. Hirayama K. Miyano H. (2009). Precolumn derivatization reagents for high-speed analysis of amines and amino acids in biological fluid using liquid chromatography/electrospray ionization tandem mass spectrometry. *Rapid. Commun. Mass Spectrom.* 23 1483–1492. 10.1002/rcm.4026 19350529

[B36] Simeone T. A. Simeone K. A. Stafstrom C. E. Rho J. M. (2018). Do ketone bodies mediate the anti-seizure effects of the ketogenic diet? *Neuropharmacology* 133 233–241. 10.1016/j.neuropharm.2018.01.011 29325899 PMC5858992

[B37] Sonnewald U. (2014). Glutamate synthesis has to be matched by its degradation - where do all the carbons go? *J. Neurochem.* 131 399–406. 10.1111/jnc.12812 24989463

[B38] Sperringer J. E. Addington A. Hutson S. M. (2017). Branched-chain amino acids and brain metabolism. *Neurochem. Res.* 42 1697–1709. 10.1007/s11064-017-2261-5 28417264

[B39] Thio L. L. Erbayat-Altay E. Rensing N. Yamada K. A. (2006). Leptin contributes to slower weight gain in juvenile rodents on a ketogenic diet. *Pediatr. Res.* 60 413–417. 10.1203/01.pdr.0000238244.54610.27 16940251

[B40] Wang T. Yao W. He Q. Shao Y. Zheng R. Huang F. (2018). L-leucine stimulates glutamate dehydrogenase activity and glutamate synthesis by regulating mTORC1/SIRT4 pathway in pig liver. *Anim. Nutr.* 4 329–337. 10.1016/j.aninu.2017.12.002 30175263 PMC6116330

[B41] Wiemer-Kruel A. Haberlandt E. Hartmann H. Wohlrab G. Bast T. (2017). Modified Atkins diet is an effective treatment for children with Doose syndrome. *Epilepsia* 58 657–662. 10.1111/epi.13701 28229464

[B42] Wilder R. M. (1921). The effects of ketonemia on the course of epilepsy. *Mayo Clin. Proc.* 2 307–308.

[B43] Wilkinson D. J. Bukhari S. S. I. Phillips B. E. Limb M. C. Cegielski J. Brook M. S. (2018). Effects of leucine-enriched essential amino acid and whey protein bolus dosing upon skeletal muscle protein synthesis at rest and after exercise in older women. *Clin. Nutr.* 37(6 Pt A), 2011–2021. 10.1016/j.clnu.2017.09.008 29031484 PMC6295981

[B44] Woodyatt R. T. (1921). Objects and method of diet adjustment in diabetes. *Arch. Intern. Med.* 28:125. 10.1001/archinte.1921.00100140002001

