# Supplementary material for: Leucine-Enriched Essential Amino Acids Enhance the Antiseizure Effects of the Ketogenic Diet in Rats
Source: Front Neurosci. 2021 Mar 19;15:637288. doi: 10.3389/fnins.2021.637288 (PMC8017216; doi:10.3389/fnins.2021.637288)
Supplement: Supplementary file 1 [file Data_Sheet_1.PDF]

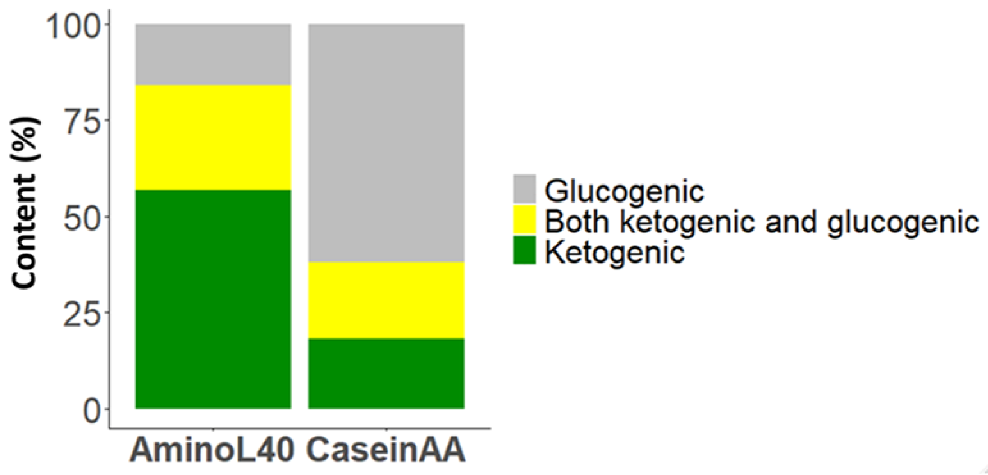

Supplementary Fig. S1
Content ratio of ketogenic and glucogenic amino acids in the AminoL40 and CaseinAA mixtures.

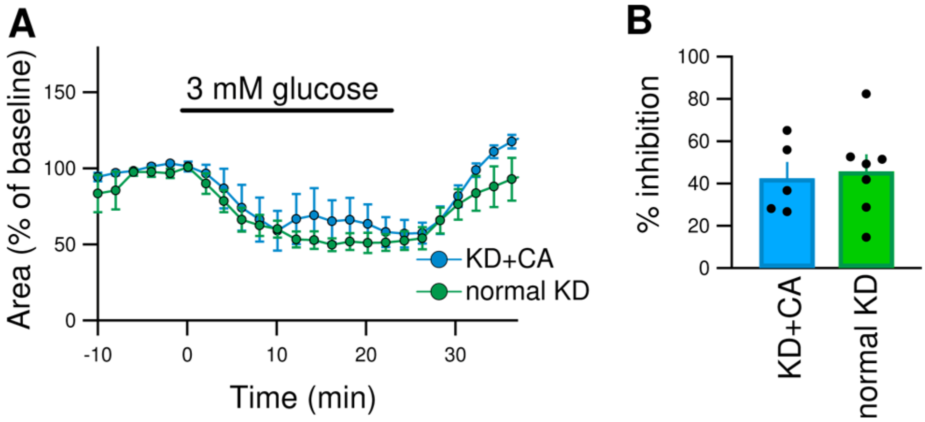

Supplementary Fig. S2

(A) Time course of the effect of a reduced glucose concentration (3 mM glucose) for 25 min on the area of bicuculline-induced bursting (% of baseline) in the slices from KD+CA-fed and normal KD-fed rats (A2) Summary of inhibition of the area of seizure-like bursting in slices from KD+CA-fed and normal KD-fed rats in the presence of 3 mM glucose. $t(10) = 0.2869$, $P = 0.7801$; unpaired *t*-test; KD+CA, n = 5; normal KD, n=7. The same data for KD+CA as in Fig. 3B and 3C. A subset of data for the normal KD was modified from a previous report (Kawamura et al. 2014).

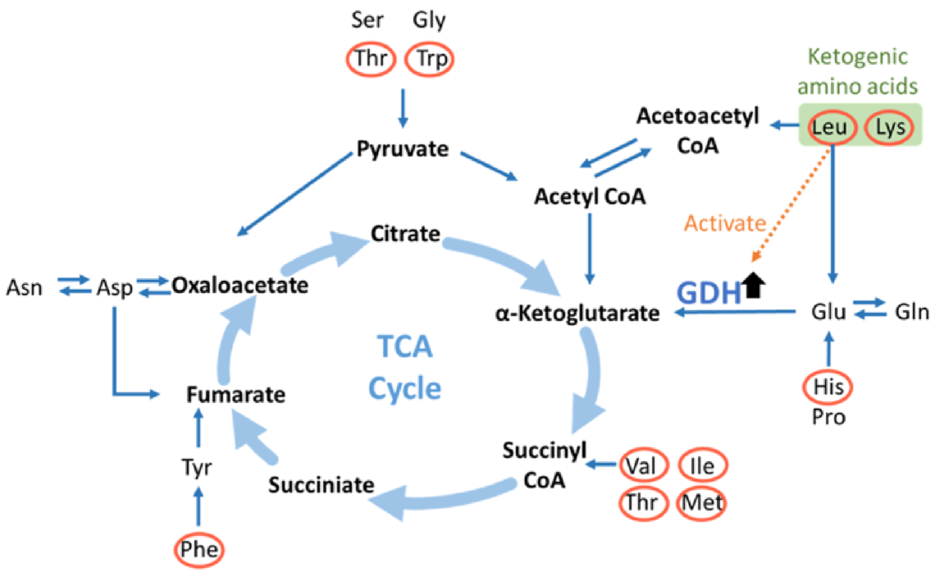

Supplementary Fig. S3

Fates of amino acids entering the TCA cycle. The red circles indicate amino acids included in AminoL40.
