# Supplementary material for: Leucine-Enriched Essential Amino Acids Enhance the Antiseizure Effects of the Ketogenic Diet in Rats
Source: Front Neurosci. 2021 Mar 19;15:637288. doi: 10.3389/fnins.2021.637288 (PMC8017216; doi:10.3389/fnins.2021.637288)
Supplement: Supplementary file 2 [file Table_1.DOC]

**Table S1: Diet composition**

| Ketogenic diet (g) | KD | KD+CA | KD+3.3% AL | KD+2.5% AL |
| --- | --- | --- | --- | --- |
| Lard | 712.5 | 689 | 689 | 694.7 |
| Butter | 299.3 | 289.4 | 289.4 | 291.8 |
| Corn oil | 171 | 165.4 | 165.4 | 166.7 |
| Casein | 142.5 | 137.8 | 137.8 | 138.9 |
| AminoL40 mixture |  |  | 49.5 | 37.5 |
| Casein mimic AA mixture |  | 49.5 |  |  |
| Cellulose | 75 | 72.5 | 72.5 | 73.1 |
| AIN76A vitamin mix | 31.4 | 30.3 | 30.3 | 30.6 |
| AIN76A mineral mix | 57 | 55.1 | 55.1 | 55.6 |
| Dextrose (D-glucose) | 11.4 | 11 | 11 | 11.1 |
| Choline bitartrate | 3 | 2.9 | 2.9 | 2.9 |
| Tert-butylhydroquinone | 0.021 | 0.02 | 0.02 | 0.02 |
| Total | 1503 | 1503 | 1503 | 1503 |
| Ketogenic ratio (KR)* | 5.3 | 4.8 | 4.8 | 4.9 |

| Nonketogenic diet (g) | CD+3.3% AL  (AIN-93G+3.3% AminoL40) |
| --- | --- |
| Corn starch | 1590 |
| Milk casein | 800.0 |
| Pregelatinized cornstarch | 528.0 |
| Granulated sugar | 400.0 |
| Soybean oil | 280.0 |
| Cellulose | 200.0 |
| AIN93G mineral mix | 140.0 |
| AIN93G vitamin mix | 40.0 |
| L-cystine | 12.0 |
| Choline bitartrate | 10.0 |
| Tert-butylhydroquinone | 0.056 |
| AminoL40 mixture | 132.0 |
| Total | 4132 |

*The KR was calculated using Woodyatt’s formula (Woodyatt, 1921), which considers the ketogenic macronutrients represented by portions of protein and fat versus glucogenic macronutrients instead of calculating the simple KR using fat (protein+ carbohydrate).

**Table S2: Amino acid mixture composition.**

| CaseinAA mixture  (%) | |  | AminoL40 mixture (%) | |
| --- | --- | --- | --- | --- |
| His | 2.71 |  | Leu | 40 |
| Ile | 4.74 |  | Lys.HCl | 16.7 |
| Leu | 8.67 |  | Val | 11 |
| Lys.HCl | 9.4 |  | Ile | 10.6 |
| Met | 2.59 |  | Thr | 9.27 |
| Phe | 4.79 |  | Phe | 6.73 |
| Thr | 4.06 |  | Met | 3.27 |
| Trp | 1.16 |  | His.HCl | 1.7 |
| Val | 6.11 |  | Trp | 0.7 |
| Ala | 2.72 |  | Total | 100 |
| Arg | 3.5 |  |  |  |
| Asn.H2O | 3.83 |  |  |  |
| Asp | 3.37 |  |  |  |
| Cystine | 0.54 |  |  |  |
| Gln | 9.77 |  |  |  |
| Glu | 9.77 |  |  |  |
| Gly | 1.72 |  |  |  |
| Pro | 9.99 |  |  |  |
| Ser | 5.39 |  |  |  |
| Tyr | 5.17 |  |  |  |
| Total | 100 |  |  |  |

**Table S3: Concentrations of amino acids in plasma.**

| **Group** | **CD** | **KD** | **KD+CA** | **KD+2.5% AL** | **KD+3.3% AL** |
| --- | --- | --- | --- | --- | --- |
| αABA | 5.56±0.831 ^a^ | 41±4.76 ^b^ | 51.7±6.47 ^c^ | 33.6±3.72 ^b^ | 34.6±6.11 ^b^ |
| Ala | 640±70.2 ^a^ | 404±49 ^b^ | 560±94.6 ^a^ | 397±71.5 ^b^ | 403±41.3 ^b^ |
| Arg | 163±14.2 ^a^ | 152±36 ^a^ | 143±16.9 ^a^ | 150±27.9 ^a^ | 151±25 ^a^ |
| Asn | 66.7±2.43 ^a^ | 86.4±9.84 ^ab^ | 95.2±13.9 ^b^ | 74.5±8.01 ^a^ | 78.6±9.54 ^ab^ |
| Asp | 10.2±1.17 ^ab^ | 11.5±4.96 ^ab^ | 14.8±2.27 ^a^ | 9.77±2.97 ^ab^ | 8.63±1.3 ^b^ |
| Cit | 121±22.1 ^a^ | 138±30.9 ^a^ | 130±13.4 ^a^ | 136±18.6 ^a^ | 134±14.1 ^a^ |
| Cys2 | 47.3±6.97 ^a^ | 10.6±1.01 ^b^ | 12.3±2.76 ^b^ | 8.71±2.2 ^b^ | 11.7±2.55 ^b^ |
| EtOH NH2 | 13.5±1.46 ^a^ | 12.2±0.716 ^a^ | 13.1±1.88 ^a^ | 12.3±0.594 ^a^ | 13.3±1.6 ^a^ |
| Gln | 694±57.9 ^a^ | 996±158 ^b^ | 1030±84.7 ^b^ | 930±142 ^ab^ | 833±62 ^ab^ |
| Glu | 92.3±19.1 ^a^ | 117±22.1 ^a^ | 125±14.1 ^a^ | 115±41.2 ^a^ | 96.1±9.67 ^a^ |
| Gly | 351±46 ^a^ | 226±13.7 ^b^ | 199±25.2 ^b^ | 188±23.7 ^ab^ | 211±29.6 ^b^ |
| His | 78.6±1.61 ^a^ | 171±22.4 ^bc^ | 181±39.7 ^c^ | 130±8.73 ^bd^ | 126±10.5 ^d^ |
| HyPro | 81.4±9.91 ^a^ | 28±4.55 ^b^ | 35.3±6.48 ^b^ | 33.7±4.61 ^b^ | 37.5±5.22 ^b^ |
| Ile | 96.4±6.15 ^a^ | 106±20.5 ^a^ | 119±19.5 ^a^ | 103±12 ^a^ | 99.2±13.1 ^a^ |
| Leu | 154±9.95 ^a^ | 162±32.6 ^a^ | 197±43.5 ^a^ | 194±40.9 ^a^ | 181±59.4 ^a^ |
| Lys | 350±79.3 ^a^ | 852±183 ^b^ | 998±347 ^b^ | 1090±123 ^b^ | 1050±120 ^b^ |
| Met | 61.1±2.63 ^a^ | 29.4±1.93 ^b^ | 38±8.84 ^b^ | 31.1±4.46 | 34.2±4.76 |
| Orn | 54.2±5.73 ^a^ | 114±29 ^b^ | 94.6±13.3 ^b^ | 96.8±13.5 ^b^ | 82.9±13.6 ^ab^ |
| Phe | 62.8±2.93 ^a^ | 44±9.12 ^b^ | 55.8±5.59 ^ab^ | 47.8±7.14 ^b^ | 44.8±7.28 ^b^ |
| Pro | 187±24 ^a^ | 276±88.7 ^ab^ | 408±96.3 ^b^ | 270±15.8 ^ab^ | 298±94.8 ^ab^ |
| Sar | 6.6±0.429 ^a^ | 5.19±0.719 ^ab^ | 4.8±1.15 ^b^ | 3.12±0.548 ^c^ | 3.12±0.502 ^c^ |
| Ser | 233±20.3 ^a^ | 659±100 ^b^ | 640±40.7 ^bc^ | 565±59.7 ^bc^ | 537±41.6 ^c^ |
| Tau | 218±34.7 ^a^ | 36.1±4.93 ^b^ | 30.1±4.37 ^b^ | 27.5±1.61 ^b^ | 28.4±4.74 ^b^ |
| Thr | 272±44.8 ^a^ | 297±64.9 ^a^ | 549±61.1 ^b^ | 1090±86.4 ^c^ | 1380±71.6 ^d^ |
| Trp | 98.9±7.11 ^a^ | 54.7±9.5 ^b^ | 84.5±18.1 ^a^ | 61.6±7.02 ^bc^ | 80.1±5.54 ^ac^ |
| Tyr | 70.6±6.45 ^a^ | 37.7±11.6 ^b^ | 71.4±23.2 ^a^ | 41.3±6.47 ^bc^ | 54.3±14.1 ^abc^ |
| Val | 208±9.83 ^a^ | 228±52.3 ^a^ | 283±58.3 ^a^ | 220±18.9 ^a^ | 218±23.8 ^a^ |
| 1-MeHis | 6.89±0.219 ^a^ | 5.52±0.214 ^a^ | 5.88±0.983 ^a^ | 5.34±0.416 ^a^ | 5.56±0.19 ^a^ |

Unit: µM; mean±SD; values above the upper limit of quantification were included. The difference in the mean was not statistically significant (P>0.05) between the groups with the same alphabetical symbols and was statistically significant (P≤0.05) between two groups with different symbols.

**Table S4: Concentrations of amino acids in the hippocampus.**

| **Group** | **CD** | **KD** | **KD+CA** | **KD+2.5% AL** | **KD+3.3% AL** |
| --- | --- | --- | --- | --- | --- |
| αAAA | 19±0.885 ^a^ | 27.3±3.3 ^ab^ | 24.8±7.75 ^ab^ | 29.1±5.07 ^b^ | 25.4±4.24 ^ab^ |
| αABA | 5.25±0.642 ^a^ | 30.4±5.52 ^b^ | 31.9±4.89 ^b^ | 22.3±3.21 ^c^ | 21.7±3.25 ^c^ |
| Ala | 823±81.5 ^a^ | 909±119 ^a^ | 904±36.4 ^a^ | 843±121 ^a^ | 778±9.14 ^a^ |
| Arg | 86.9±13 ^a^ | 48.4±9.71 ^b^ | 40.4±5.72 ^b^ | 40.7±7.44 ^b^ | 41.8±5.56 ^b^ |
| Asn | 96.2±8.66 ^a^ | 120±16.7 ^b^ | 110±5.64 ^ab^ | 123±12 ^b^ | 117±7.04 ^ab^ |
| Asp | 1430±114 ^a^ | 1520±180 ^a^ | 1550±60.4 ^a^ | 1540±59.7 ^a^ | 1440±107 ^a^ |
| Car | 7.35±0.616 ^a^ | 12.7±2.47 ^b^ | 8.14±1.49 ^a^ | 8.8±1.06 ^a^ | 8.23±1.78 ^a^ |
| Cit | 43.2±0.714 ^a^ | 48.6±12.6 ^a^ | 38.3±1.5 ^a^ | 36.4±6.53 ^a^ | 37.5±10.8 ^a^ |
| Cysthi | 22.9±7.42 ^a^ | 43.1±1.86 ^b^ | 28.8±9.49 ^ab^ | 32.8±7.11 ^ab^ | 29.9±10.2 ^ab^ |
| EtOH NH2 | 228±4.27 ^a^ | 271±38.1 ^ab^ | 277±25.4 ^ab^ | 264±21.8 ^ab^ | 285±35.6 ^b^ |
| GABA | 1190±59.1 ^a^ | 1430±220 ^a^ | 1470±291 ^a^ | 1430±242 ^a^ | 1500±253 ^a^ |
| Gln | 3530±285 ^a^ | 4980±434 ^b^ | 4520±409 ^bc^ | 4050±171 ^ac^ | 4270±298 ^c^ |
| Glu | 9590±890 ^a^ | 10400±193 ^a^ | 10700±818 ^a^ | 10200±276 ^a^ | 10400±499 ^a^ |
| Gly | 691±41.8 ^a^ | 956±110 ^b^ | 856±43 ^ab^ | 945±126 ^b^ | 858±57.5 ^ab^ |
| His | 72.3±4.26 ^a^ | 190±36.3 ^b^ | 133±17.6 ^c^ | 106±12.7 ^ac^ | 109±12.5 ^ac^ |
| HyPro | 36.8±4.38 ^a^ | 13.7±4.14 ^b^ | 16.1±3.91 ^b^ | 15.5±3.63 ^b^ | 14.1±4.17 ^b^ |
| Ile | 37.2±1.71 ^a^ | 52.2±18.4 ^a^ | 45.1±5.37 ^a^ | 39.8±3.23 ^a^ | 37.9±2.52 ^a^ |
| Leu | 73.6±2.93 ^a^ | 91.6±23.9 ^a^ | 90.1±11.1 ^a^ | 90.6±13 ^a^ | 77.8±11.9 ^a^ |
| Lys | 151±31.4 ^a^ | 215±40.5 ^ab^ | 213±58.3 ^ab^ | 234±26.4 ^b^ | 230±15 ^b^ |
| Met | 37.7±3.44 ^a^ | 22.7±1.83 ^b^ | 22.7±3.35 ^b^ | 18.6±1.66 ^b^ | 20.3±2.02 ^b^ |
| Orn | 10.9±2.13 ^a^ | 10.8±2.97 ^#a^ | 7.57±1.1 ^b^ | 7.6±1.38 ^ab^ | 6.97±0.612 ^c^ |
| Phe | 38.6±3.51 ^a^ | 39.3±9.13 ^a^ | 35.3±1.38 ^ab^ | 31.5±3.47 ^ab^ | 28.5±2.15 ^b^ |
| Pro | 64.4±5.38 ^a^ | 75.1±18.7 ^a^ | 66.1±4 ^a^ | 66.9±7.69 ^a^ | 60.9±4.42 ^a^ |
| Sar | 0.438±0.0708 ^a^ | 0.848±0.546 ^a^ | 0.572±0.11 ^a^ | 0.688±0.484 ^a^ | 0.468±0.138 ^a^ |
| Ser | 759±35.8 ^a^ | 1380±247 ^b^ | 1120±90.5 ^c^ | 1030±30.7 ^c^ | 1000±99.9 ^ac^ |
| Tau | 1100±91 ^a^ | 1210±456 ^a^ | 1390±478 ^a^ | 1180±351 ^a^ | 1460±463 ^a^ |
| Thr | 475±59.7 ^a^ | 495±54.4 ^ab^ | 710±96.2 ^b^ | 1210±96.2 ^c^ | 1420±184 ^c^ |
| Trp | 15.7±1.74 ^a^ | 19±4.65 ^a^ | 18.9±2.47 ^a^ | 15.8±1.19 ^a^ | 18.2±2 ^a^ |
| Tyr | 53.9±7.35 ^a^ | 42.8±10.8 ^ab^ | 55.9±13.9 ^a^ | 34.1±3.75 ^b^ | 42.3±6.96 ^ab^ |
| Val | 101±5.14 ^ab^ | 123±25.7 ^a^ | 121±13.3 ^a^ | 92.7±5.9 ^b^ | 85.2±7.98 ^b^ |
| 1-MeHis | 3.41±0.758 ^a^ | 4.62±0.851 ^a^ | 3.04±0.871 ^a^ | 2.99±0.999 ^a^ | 3.84±1.31 ^a^ |
| 3-MeHis | 1.86±0.219 ^a^ | 1.74±0.252 ^ab^ | 1.42±0.11 ^ab^ | 1.35±0.0508 ^b^ | 1.65±0.383 ^ab^ |

Unit: µM; mean±SD; value above the upper limit of quantification were included. #, removing outliers (n=1). The difference in the mean was not statistically significant (P>0.05) between groups with the same alphabetical symbols and was statistically significant (P≤0.05) between two groups with different symbols.
